# Human and Artificial Intelligence (AI) Analysis of Patient Experiences of Periodontal Graft Surgery

**DOI:** 10.3390/dj14020127

**Published:** 2026-02-23

**Authors:** William W. N. Mak, Timothy Budden, Sushil Kaur, Maurice J. Meade

**Affiliations:** 1 Periodontics Unit, Adelaide Dental School, University of Adelaide, Adelaide, SA 5005, Australia; william.mak@student.adelaide.edu.au (W.W.N.M.); sushil.kaur@adelaide.edu.au (S.K.); 2 Adelaide Dental School, University of Adelaide, Adelaide, SA 5005, Australia; a1192918@adelaide.edu.au; 3 Orthodontic Unit, Adelaide Dental School, University of Adelaide, Adelaide, SA 5005, Australia

**Keywords:** patient experience, periodontal surgery, social media, YouTube, artificial intelligence, qualitative analysis, quality of information

## Abstract

**Background/Objectives**: The prominent role the internet plays in being a source of dental information prompts qualitative evaluation of relevant online content. This study aimed to explore patients’ experience regarding periodontal graft surgery communicated through the social media platform YouTube. **Methods**: An initial YouTube search using the term “gum surgery experience” retrieved 40 videos. Graft surgery was the most frequently discussed procedure, and 19 relevant videos were included in the qualitative analysis. Video content was analysed using a combined human-centered and artificial intelligence (AI)–assisted approach. AI-supported analysis of viewer comments was conducted using ChatGPT-4 and Gemini-1.5 Pro. Themes generated by human and AI analyses were compared. **Results**: Nine key themes were identified from the 19 videos that satisfied selection criteria. Most themes were similar between human and AI analyses, with six overlapping and three unique. The most frequently coded theme was post-operative recovery (*n* = 177), with pain, work absence, eating difficulties, and disrupted oral hygiene commonly reported. Patient-clinician relationships were frequently highlighted, with mixed experiences regarding communication and trust. Positive experiences were reported more frequently than negative. Comment analysis revealed varied audience engagement and sentiments, emphasizing concerns about pain, recovery, and procedural anxiety. **Conclusions**: Key themes related to patient experiences were identified, notably concerns regarding post-operative recovery and patient-clinician relationships. Challenges in finding information prior to having surgeries motivated patients to provide support and advice on YouTube, emphasizing the need for patient-centered resources and effective patient-clinician communication. Integrating human and AI methods in qualitative analysis was efficient and insightful, with AI supplementing but not substituting human research.

## 1. Introduction

Data related to patient experience are an increasingly useful source of information to gain valuable insight into a patient’s direct encounter with medical and dental treatment. Assessing patient experience allows for improvement in service, clinical outcomes, and reduction in costs [1]. This is possible through the identification of problems in the care process, which include the coordination of care, the care environment and the provision of treatment [2]. All National Health Service (NHS) funded services in England have been mandated to provide the opportunity for patients to share their experience and feedback since April 2013 via the Friends and Family Test (FFT) [3]. As in the UK, the United States (US) healthcare system places significant emphasis on patient feedback through surveys like the Hospital Consumer Assessment of Healthcare Providers and Systems (HCAHPS) [4]. Traditionally, patient experiences have been collected using conventional tools, such as paper based questionnaires [5]. However, with the increase in popularity of the internet and social media, patients are using online platforms to share their experiences publicly [5]. In fact, social media has become deeply ingrained in the American culture, with 76% of online adults in the US using social media [6].

One of the methods that can be utilized by patients to share their treatment experiences online includes vlogs, which are a form of online content where individuals share video recordings of their activities, thoughts, experiences, or tutorials through social media platforms such as YouTube (San Bruno, CA, USA). YouTube’s superiority over other social media platforms is evident in its capacity to host richer content, such as videos. Shahbaznezhad and colleagues asserted that videos are more impactful in social media communication compared to those with lower rich content [7]. These videos allow users to share their experiences in detail because there is no limit to the length of each video. The US has the second-largest YouTube audience in the world, with approximately 254 million viewers as of October 2025 [8]. This significant user base reflects the platform’s extensive reach and influence in the US, making it a crucial medium for sharing and accessing information. Since the launch of YouTube, vlogs have increasingly gained popularity among internet users [9], with 42% of users watching vlogs each month according to the report by Global Web Index [10].

Artificial intelligence (AI) is defined as the simulation of human intelligence in machines that are programmed to think and learn. The integration of AI into healthcare research has introduced new ways to analyze patient feedback and improve care [11]. AI systems can process large amounts of data, recognize patterns, and make decisions with minimal human input [12]. Specifically, AI’s ability to process and analyze textual data, such as patient comments and feedback, enables researchers to gain deeper insights into patient experiences and identify trends that might not be apparent through human analysis [12].

It has also been shown that information from interactions on social media platforms, including user comments, can serve as a potentially rich source of qualitative data, offering valuable insights into users’ viewpoints, emotions, and personal experiences [13]. YouTube is as a prominent platform characterized by its extensive repository of public comments and active user engagement [14]. However, manual analysis of these data is both time-consuming and prone to subjective interpretation, presenting a considerable obstacle for researchers in this domain [15]. Consequently, recent advancements in AI have sparked optimism regarding its potential application in qualitative research [15]. In 2023, Hamilton and colleagues explored the feasibility of harnessing AI, specifically ChatGPT (San Francisco, CA, USA), to complement traditionally human-driven tasks in qualitative research analysis, such as processing textual data and generating qualitative themes [12]. The study reported that AI offered the potential to identify themes that might not be apparent to human coders [12]. AI’s capacity to identify patterns, themes, and sentiments within textual data provides significant advantages in processing, analyzing, integrating, and triangulating data [15].

Understanding and incorporating patient feedback is particularly crucial in the context of periodontal surgery, where patient anxiety and the need for clear communication are heightened. Periodontal surgery includes interventions such as access flap surgery, resective flap surgery and regenerative surgery [16]. Additional surgical procedures commonly carried out include canine exposure surgery to facilitate orthodontic treatment, dental implant surgery, crown lengthening and frenectomies. Treatment of periodontal disease begins with scaling and root debridement, which is a non-surgical procedure that involves thorough removal of plaque, calculus, and bacteria from the root surfaces of teeth [17]. The surgical phase of periodontal treatment is usually performed after re-evaluation of the non-surgical debridement, with the aim of treating periodontal sites that are not responding to non-surgical approach [17]. In a market analysis by Market Research Future, the US dominates the global periodontal soft tissue grafting market due to its substantial elderly population and high levels of healthcare expenditure [18]. In 2023, the soft tissue grafting market was valued at USD 0.45 billion [18]. The industry is expected to grow from USD 0.50 billion in 2024 to USD 0.94 billion by 2032, with a compound annual growth rate (CAGR) of 7.33% over the forecast period from 2024 to 2032 [18]. Increased patient education regarding the surgical procedure and effective communication with clinicians contribute to a more positive attitude towards the treatment and greater overall satisfaction with the process. Additionally, this approach fulfils the requirements for obtaining valid consent [19,20]. Olson and Laskin, for example, demonstrated that patients experienced reduced psychological trauma and higher satisfaction levels when provided with detailed preoperative information about their surgical procedure [21].

To date, research examining patients’ perceptions of periodontal surgery as shared on social media remains limited, and studies leveraging artificial intelligence (AI) to systematically analyse such patient-generated content are particularly scarce. As social media increasingly shapes patient understanding and expectations of dental procedures, there is a clear need for rigorous methods that can capture both the experiential and emotional dimensions of these narratives at scale. Gaining insight into patients’ treatment experiences may enable dental professionals to better address patient concerns and identify unmet informational needs. Therefore, the present study aimed to evaluate patients’ experiences with periodontal graft surgery by analysing YouTube video content and viewer comments using a combined human coding and AI-assisted analytical approach, thereby offering a novel and comprehensive perspective on patient-reported experiences in the digital health information landscape.

## 2. Materials and Methods

The present study involves qualitative analysis of online information that is publicly available. As such, ethical approval was not required.

### 2.1. Search Strategy

The purpose of the search strategy was to identify YouTube videos relevant to periodontal surgery. With the aid of Google Trends, the term ‘Gum Surgery’ was the most widely used relevant searched phrase over the previous 5 years (2017–2022). The search term “gum surgery experience” was entered into the YouTube search bar between 13 March 2024 and 20 May 2024 to identify videos documenting patients’ personal experiences with periodontal surgery. From the videos retrieved, the most frequently discussed procedure (‘gingival graft’) was chosen for qualitative analysis. To minimize the influence of previous search histories, the searches were conducted without signing into any YouTube user account and with the YouTube watch history turned off. This strategy aligns with the findings from the Massachusetts Institute of Technology (MIT) Tech Review, which stated that 70% of what people watch on YouTube is suggested by the platform’s recommendation programme [22]. The unique resource locator (URL) of each video was recorded on an Excel (Microsoft Corporation, Redmond, WA, USA) spreadsheet. Videos were excluded if they were: (1) not in the English language; (2) duplicate entries; (3) uploaded by dental professionals; (4) not relevant to actual patient experience and (5) not relevant to gingival graft surgery.

### 2.2. Qualitative Assessment

#### 2.2.1. Human-Centered Approach

The “Show Transcript” function on the YouTube platform was utilized to generate a full transcript of the spoken content in the videos. These transcripts were transferred to a Microsoft Word document (Microsoft Corporation, Redmond, WA, USA) for initial coding. The coding process involved two stages: initial and axial. The in vivo coding approach (using participants’ own words or phrases as codes) was adopted for initial coding where a list of open codes was documented. During this stage, the video transcripts were examined line by line to capture information and assign codes to these pieces. The purpose of open coding was to generate an initial list of codes that represent different themes in the data. These codes were then further refined and grouped by the implementation of axial coding (deriving subcategories from the initial list of open codes). The primary objective was to identify relationships among the categories noted in the initial open coding phase. This was followed by comparison and grouping of codes for inductive content analysis (ICA), using the steps outlined by Vears and Gillam (2022) [23]. This study also utilized Google’s AI software, Gemini-1.5 Pro (Google DeepMind, New York, NY, USA), to highlight recurring key points that could have been missed by the authors. The URL for each video was copied over to Gemini with specific prompts to generate a summary of each video and to identify recurring concepts, themes, key points, and sentiments. The authors then reviewed these recurring key points to identify additional codes.

The findings were recorded in a Microsoft Excel (Microsoft Corporation, Redmond, WA, USA) spreadsheet. The generated codes were combined and uploaded to a cloud-based qualitative data analysis software (Delve Coding Software, New York, NY, USA). A codebook was created using the Delve Software platform (www.delvetool.com, accessed on 15 April 2024), featuring detailed descriptions for each individual code. Pilot coding was conducted to identify and address any disagreement between two coders, WM and TB. In instances of disagreement, both coders carefully reviewed the codebook to clarify ambiguous definitions and rectify any misunderstanding through discussion. The two researchers then assigned the codes to each video transcript through the software.

**Figure 1:**
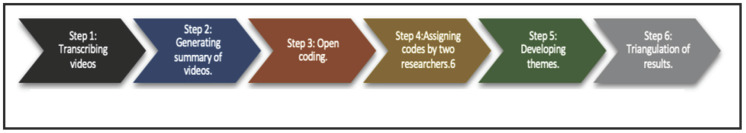
Summary of the six-step coding process.

Krippendorff’s Alpha intercoder reliability score, which measures how consistently different authors are applying codes to the video transcript, was calculated for each video transcript using the automated function of the Delve software. A mean score was reported, for which the lowest acceptable score was 0.667 [24]. Figure 1 summarizes the six-step coding process used in this qualitative research. The assigned codes were also used to develop common themes using the human-centered approach.

#### 2.2.2. AI Approach

The transcripts of the videos that satisfied inclusion criteria were uploaded to the ChatGPT-4 (OpenAI, San Francisco, CA, USA) platform with the following prompts: (i) “Act as a qualitative researcher” and (ii) “Please identify common themes from the statements.” [15].

A comparison between the human generated themes and the AI generated themes using the steps outlined by Hamilton et al. (2023) was carried out [12]. In addition, the videos underwent sentiment analysis whereby they were categorized into positive, negative, or neutral emotional tones by the lead author, WM. To enhance knowledge of the online community’s perception of periodontal grafting surgery, the comments section of the 19 videos underwent further AI analysis with the assistance of a software system (YouTube Comment Generator & Analyse) by EasyComment (Dunearn Estate, Singapore). Information generated from the analysis included:

- audience engagement score (number of viewer likes + comments/views × 1000),
- sentiment,
- emotion (represented by the most frequent emojis found in the comments),
- comments with the most likes and comments with the most replies.

**Figure 2:**
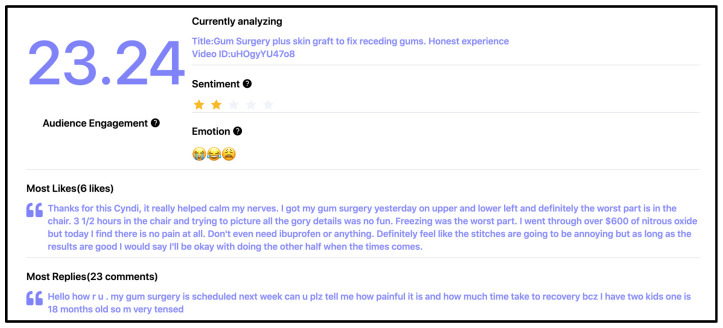
An example of the AI analysis output from the EasyComment software.

An example of a screenshot obtained from the AI analysis is illustrated in Figure 2.

## 3. Results

**Figure 3:**
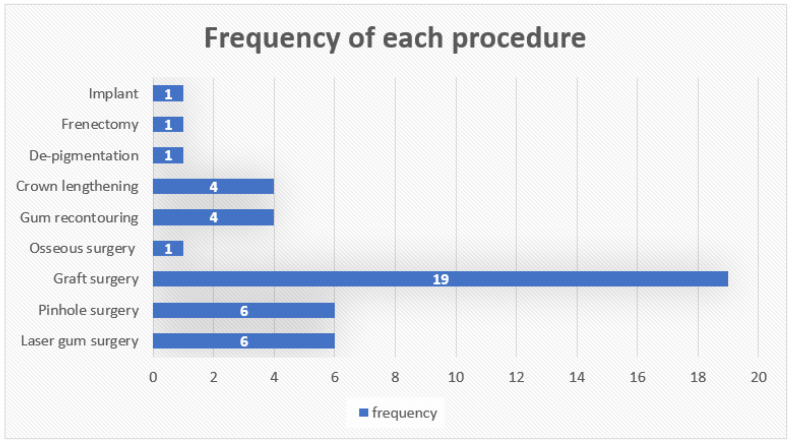
Frequency of each procedure as recorded from the YouTube videos.

From the initial search, 40 YouTube videos were retrieved. ‘Graft’ surgeries were the most discussed surgical procedure (*n* = 19) (Figure 3). Only these 19 videos were included in the qualitative analysis. Of these, eight were filmed at the dental practices where the vloggers underwent their procedures.

The median (IQR) number of views for these videos was 2900 (27,793.5) ranging from a minimum of 86 views to a maximum of 228,000 views. The Shapiro–Wilk test indicated a non-normal distribution. Ten themes were identified by the ‘human centered’ approach, and eight themes were generated by AI (ChatGPT). The main themes from the human-centered and AI generated approach were combined and reported in Table 1.

**Table 1:** Main themes according to frequency of appearance (N).

Main Themes	Frequency of Appearance (N)
Postoperative Recovery	177
Surgical Experience	44
Outcome and Satisfaction	37
Compliance	17
Pre-Surgery Expectations and Knowledge	54
Patient-clinician Interaction	57
Emotional and Psychological Impact	47
Financial Considerations	10
Recommendations and Advice	17

Refer to Table 2 for definitions and representative statements of each main theme.

**Table 2:** Definition and representative statements of main themes.

Main Theme	Representative Statement
Postoperative Recovery *Statements regarding patients’ experiences during the recovery phase.*	“Can still feel pain few days after surgery.” “Swelling next day after surgery.” “Discomfort still present after 5 days.”
Surgical Experience *Descriptions of patients’ past experiences and experiences during the surgical procedure.*	“Procedure was not too unpleasant.” “Thought surgery was no big deal, ended up being a big process.” “Numbing injection on palate hurts so bad.” “First procedure 4–5 years ago. Extremely extremely painful. Dentist recommended graft for another teeth. “
Outcome and Satisfaction *Comments on the perceived success or failure of the procedure and overall satisfaction.*	“Glad that surgery was done.” “So thrilled with the result.” “Can now smile after surgery, can eat hot and cold doesn’t bother me.”
Compliance *Statements regarding adherence to postoperative instructions and dietary restrictions.*	“Soft diet for 2 weeks after surgery. Hard having restrictions on what you can eat.” “Followed post op instructions to the letter.”
Pre-Surgery Expectations and Knowledge *Statements about patients’ knowledge and expectations before the surgery.*	“Only found out about the actual plan of surgery on the day.” “Didn’t actually say where they would even take the skin from.”
Patient-clinician Interaction *Feedback on the interactions with dental professionals, including communication and support.*	“Staff made me feel welcomed and calm.” “Doctor was never present during reviews, only dental assistants. Very frustrating.”
Emotional and Psychological Impact *Statements related to patients’ emotional responses and psychological experiences.*	“Decided not to return to the periodontist and to just let the gum graft grow by its own.” “Didn’t want to have it done again.”
Financial Considerations *Comments on the cost of the procedure and insurance coverage.*	“Extremely expensive.” “Insurance covered half of it.” “Periodontist cleans are twice the price of general dentist and finds it very expensive.”
Recommendations and Advice *Suggestions or advice provided by patients based on their experiences.*	“Periodontists are trying to make money and just to be careful.” “Recommend viewers to get it done if you needed this procedure.”

Overall, analysis of the YouTube videos revealed that post-operative recovery experiences, patient–clinician relationships, and emotional responses to periodontal graft surgery were the most prominent findings. Post-operative recovery was the most frequently coded theme, with patients commonly reporting pain, functional limitations, and disruptions to daily activities. In addition, sentiment analysis indicated that experiences were predominantly positive, despite the presence of notable negative accounts. A substantial overlap was also observed between human- and AI-generated thematic analyses, supporting the consistency of the combined analytical approach.

**Figure 4:**
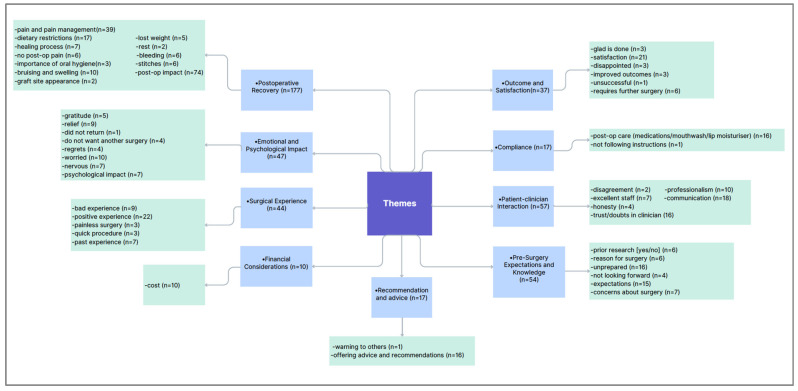
Distribution of main themes and subthemes.

The main themes were further explored through subthemes, as detailed in Figure 4. Post-operative recovery following graft surgery was the most frequently coded theme (*n* = 177). Most YouTubers (hereafter referred to as vloggers) described post-operative impacts such as pain of varying intensity, time off work, difficulty eating, and challenges in maintaining oral hygiene immediately after surgery. For example, one vlogger noted that she “had lost 5 pounds since the surgery and did not like the gum graft weight loss programme” (Video 11).

A side-by-side comparison of human versus AI generated themes are listed in Table 3. The similarities and differences between the two approaches are further summarized in Table 4. There were 6 overlapping themes and 3 unique themes. Therefore, approximately 67% of the themes were similar between the human-generated and AI-generated themes.

Sentiment analysis conducted using the human-centered approach revealed that, of the 19 videos analysed, 10 conveyed a positive emotional tone, 6 were negative, and 3 were neutral. This distribution was mirrored in the content analysis, in which positive experiences were reported more frequently than negative experiences (*n* = 22 and *n* = 9, respectively). Common positive descriptors included “painless experience,” “not too unpleasant,” and “better than expected,” and five vloggers stated that they would recommend the procedure to others. In contrast, negative experiences were characterized by terms such as “unpleasant,” “bad experience,” and “painful procedure,” often accompanied by references to surgical challenges.

Patient–clinician relationships also emerged as a key theme. The majority of vloggers reported positive interactions with their clinicians, particularly highlighting effective communication. Clinicians were frequently described as having good communication skills (*n* = 10) and providing clear explanations (*n* = 2). However, two vloggers perceived their clinicians as dishonest, which negatively affected their trust and overall experience.

Several vloggers reported actively seeking information about periodontal graft surgery prior to treatment and commonly cited a lack of relevant and reassuring online resources. One vlogger remarked, “I could barely find anything online that would give me comfort or ease my anxiety” (Video 18). As a result, many vloggers stated that their motivation for sharing their experiences was to support others undergoing similar procedures. By contrast, one video (Video 5) adopted a cautionary stance, alleging that periodontists prioritized profit over patient welfare.

An overview of YouTube comment analysis is presented in Table 5. Among videos with available data, the median (IQR) audience engagement score was 19.83 (22.91), with a maximum score of 64.14. Sentiment analysis of comments revealed varied reactions, with two videos eliciting neutral overall responses, three prompting predominantly negative reactions, and two receiving positive overall sentiment. Highly engaged comments frequently expressed concerns related to pain, recovery, fear, and anxiety following graft surgery. Commenters also shared personal surgical experiences, discussed procedural details, exchanged coping strategies, raised financial concerns, and offered emotional support and encouragement to others.

The inter-coder reliability of the 19 videos, assessed by the mean (SD) Krippendorff’s Alpha, was 0.625 (0.112).

## 4. Discussion

The aim of this study was to gain insight into patients’ experiences of periodontal graft surgery as communicated through a social media platform using both human-centered and AI-assisted analyses. Based on the thematic analysis, nine main themes were identified through the combined human and AI methodology. These themes highlighted vloggers’ intentions to share their personal experiences following periodontal graft surgery, often with the aim of offering support to individuals considering similar procedures. Several vloggers reported difficulty finding adequate information or videos that provided reassurance prior to surgery. This lack of accessible information may have motivated them to produce videos from their own perspectives to help others make informed decisions. This finding aligns with the study by Liu et al. (2013), which identified patient connections—such as those formed between vloggers and their audiences—as a significant source of social support and a form of self-therapy for health vloggers [25].

The present study suggests that YouTube is a valuable social media platform for sharing treatment experiences from the patient’s perspective. One key advantage of YouTube is that, unlike other social media platforms such as Instagram (Menlo Park, CA, USA) and TikTok (Culver City, CA, USA), there is no restriction on video length. By contrast, Instagram and TikTok support shorter videos, up to 60 s and 10 min, respectively. This allows vloggers to share more detailed accounts of their experiences on YouTube. Based on the videos analysed in this study, vloggers often included their experiences before, during, and after the procedure. However, a data report by Lindner (2024) found that over 60% of viewers preferred concise videos, with most losing interest after three minutes [26]. Ho and colleagues (2018) similarly emphasized the importance of balancing conciseness with the provision of sufficient information [27].

Social media platforms may also serve as valuable tools for assessing patients’ opinions and sentiments, complementing traditional methods of patient feedback collection such as surveys, focus groups, and interviews [28]. Collecting patient experience data through YouTube allows individuals to express their views freely while potentially minimizing the Hawthorne effect associated with more controlled research settings. The Hawthorne effect refers to behavioural changes that occur when individuals are aware they are being observed or evaluated [29]. A systematic review by McCambridge et al. (2014) supported this observation, demonstrating evidence of the Hawthorne effect in most studies analysed, particularly when participants were aware of their involvement through interviews or questionnaires [30]. Nevertheless, a potential challenge of sharing content on YouTube is exposure to negative or impolite audience comments [31]. To better understand audience perceptions of graft surgery, the present study employed AI-assisted analysis of YouTube comments. The primary motivations for comment engagement were information sharing, information seeking, and social interaction, findings that are consistent with a recent survey-based study on YouTube participation and information consumption [14]. However, it should be noted that some videos had the comments function disabled and were therefore excluded from this analysis.

Although some information shared by vloggers was inaccurate or influenced by personal bias—such as claims regarding pregnancy causing gingival recession or the effectiveness of professional cleaning every three months in preventing recession—most videos reflected patients’ overall experiences, treatment attitudes, and perceptions of care. This finding is consistent with a study by Nguyen and Allen (2017), which reported that while approximately one-third of testimonial videos on YouTube contained inaccuracies, many still offered valuable insights into patient perspectives and experiences [32]. Their study also found that most patient experiences shared on YouTube were perceived as positive, a finding echoed in the present study, where positive experiences were expressed more frequently than negative ones.

Post-operative quality of life emerged as an important theme, with recovery-related challenges significantly affecting patients’ well-being. Post-operative pain often limited individuals’ abilities to perform routine activities, while dietary restrictions negatively impacted nutrition and enjoyment of food. These findings align with existing research identifying fear of oral pain as a primary barrier to seeking dental treatment [33]. Videos containing such information may potentially deter patients from undergoing similar procedures. Concerns were also raised regarding post-operative expectations. One vlogger reported not anticipating the need for stitches, suggesting possible deficiencies in pre-operative communication, patient recall, or alignment of expectations. A Slovenian study from 2011 similarly found that patients often struggled to recall medical information provided during consultations [34], and a more recent study reported poor comprehension and retention of pre-surgical information [35].

Cost was another prominent theme, with several patients expressing concern about the financial burden of periodontal graft surgery. One vlogger specifically complained about additional costs associated with dental cleaning during a follow-up appointment. Comparable findings have been reported in qualitative studies from Sweden and Republic of Korea, where patients highlighted the financial strain associated with periodontal treatment [36,37,38].

Patient–clinician relationships were discussed frequently, with many vloggers expressing satisfaction with their clinicians and clinic staff. However, two vloggers reported a loss of trust due to perceived dishonesty. One vlogger described being informed at a one-year post-operative review that further surgery might be required, leading her to question the necessity of additional treatment and ultimately not adhere to the clinician’s recommendations. She also cautioned other viewers, suggesting that periodontists may prioritize profit and advising vigilance. This response may reflect patient disappointment when treatment outcomes do not meet initial expectations [39]. These findings underscore the importance of clear, transparent communication in fostering trust. When patients receive comprehensive and realistic pre-operative information, they are more likely to experience reduced psychological distress and greater satisfaction [21].

It is essential for clinicians to be aware of the social media content their patients access. A recent systematic review reported a high prevalence of health misinformation on social media and identified YouTube as the most frequently used platform for seeking information about medical treatments [40]. Another study surveying 600 participants found that individuals with lower health literacy were less likely to trust information from health professionals, including dentists, and more likely to rely on social media sources [41]. Public mistrust has been further amplified by the COVID-19 pandemic, particularly in relation to COVID-19 vaccinations [42,43]. As a result, patients are increasingly likely to seek health information online. This study highlights key aspects of periodontal graft surgery that matter most to patients, including recovery, patient–clinician relationships, and cost. These insights have the potential to directly inform how clinicians deliver pre-surgical information. Although the findings are specific to periodontal grafting, they may be applicable to other surgical procedures and are therefore relevant to a broad range of medical and dental professionals. Policymakers may also consider the role of platforms such as YouTube in disseminating health information, particularly for individuals with lower health literacy.

The present study also demonstrated that integrating AI into qualitative analysis offers several advantages. AI enables rapid and efficient processing of large datasets and may identify themes that are not immediately apparent to human coders, supporting findings by Hamilton et al. (2023) [12]. However, AI lacks the analytical depth, contextual sensitivity, and interpretive flexibility of human researchers. Consequently, findings generated through AI-assisted analysis require careful contextualization and interpretation by human experts. Future qualitative research would benefit from integrating AI tools within a human-centered analytical framework.

In determining the sample size, the authors adopted a search strategy consistent with previous YouTube-based medical studies [44,45]. Unlike prior studies that typically analysed the first 10 or 20 videos, this study included 40 videos to ensure broader representation of available content. From this pool, 19 videos related specifically to graft surgery were selected for in-depth analysis. This targeted approach enhanced the relevance of the findings. To further strengthen methodological rigor, researcher triangulation was employed, with two researchers independently coding each transcript. This process facilitated cross-validation of coding decisions and minimized subjective bias, thereby improving the validity of the findings.

Several limitations should be acknowledged. As a dynamic platform, YouTube content varies over time, and searches conducted at different points may yield different results. The analysis was also limited to English-language videos, which may not fully represent global patient experiences. Inter-rater reliability was another limitation, as the mean Krippendorff’s Alpha (0.625) was slightly below the 0.667 threshold cited as acceptable. This may be attributed to the subjective nature of content interpretation, variability in video formats, and challenges inherent in coding user-generated content. Despite this, the level of agreement indicates moderate reliability, and the findings remain informative. Finally, potential bias may have arisen from videos filmed in dental practices (8 out of 19), where vloggers may have been more inclined to portray their experiences positively due to their association with the clinic.

In conclusion, this study provides valuable insights into patients’ experiences with periodontal graft surgery as shared on YouTube. Vloggers who encountered difficulties finding pre-surgical information often sought to support others considering similar procedures, highlighting the need for more patient-centered resources. Patient–clinician relationships emerged as central to patient satisfaction, emphasizing the importance of effective communication. Clinicians should assess patient comprehension and tailor information delivery throughout the treatment process, addressing both physical outcomes and quality-of-life impacts. The combined human and AI analytical approach offered both efficiency and depth; however, AI should be viewed as a complementary tool rather than a replacement for human expertise. Overall, YouTube represents a valuable platform for understanding patient perspectives and informing future research and clinical practice.

## Figures and Tables

**Table 3:** Side-by-side Comparison between Human and AI Generated Qualitative Themes.

Human Generated Themes	ChatGPT Generated Themes
Post-op impact ^†^	Postoperative recovery
Compliance	
Patient-clinician relationship	Patient-provider interaction
Improved quality of life (QoL) ^‡^	Outcome and satisfaction
Cost	Financial considerations
Past experience	Surgical experience
Psychological impact	Emotional and psychological impact
Positive feelings	
Negative feelings	
Absence from work	
	Pre-surgery expectations and knowledge
	Recommendations and advice

^†^ *Effects or consequences experienced by a patient following surgical procedure. Post-operative impact may include a range of physical, emotional, and practical changes or challenges experienced by the patient*. ^‡^ The impact of periodontal health on a person’s overall sense of well-being and satisfaction with various aspects of their life.

**Table 4:** Similarities and Differences between Human and AI Generated Qualitative Themes.

Similarities	
Emotional Impact	Both human and AI-generated themes addressed the emotional impact of the surgical experience, including negative and positive feelings, as well as psychological effects.
Patient-clinician Interaction	Both approaches recognized the importance of the relationship between patients and healthcare providers, albeit expressed in slightly different terms.
Financial Considerations	Both highlighted the significance of financial aspects in the context of treatment, whether it is the cost mentioned by humans or broader financial considerations by AI.
Postoperative Recovery	Both acknowledged aspects related to the postoperative phase, including impacts on daily life, psychological well-being, and absence from work.
Surgical Experience	Both recognized patients’ prior experiences as well as during the surgical procedure.
Outcome and satisfaction	Both recognised the diverse perceptions of the outcome, with some patients satisfied and others expressing disappointment or uncertainty.
Differences	
Compliance	Human-generated themes included compliance as a separate aspect, while AI-generated themes listed adherence to postoperative instructions and dietary restrictions under postoperative recovery.
Recommendations & Advice	AI-generated themes incorporated recommendations and advice for patients, which are not explicitly addressed in the human-generated themes.
Pre-Surgery Expectations & Knowledge	This aspect is mentioned only in AI-generated themes, indicating a focus on what patients anticipate before undergoing surgery and their level of understanding.

**Table 5:** Analysis of YouTube comments.

Video	No. of Comments	No. of Replies	Audience Engagement Score	Sentiment Score (out of 5) * 1: Negative; 5: Positive.	Emotions
1	83	200	23.23	3	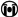 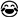 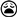
4	43	8	22.67	5	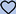 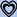 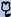
5	56	53	13.96	2	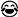 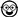 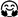
6	30	46	64.14	3	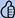 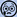 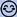
10	3	0	7.31	2	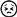 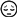
12	31	14	53.0	4	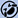 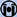 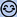
14	24	34	16.99	2	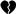 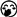 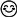
18	57	85	16.45	3	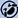 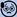 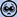

* Videos 2, 3, 7, 8, 9, 11, 13, 15, 16, 17, and 19 were not listed on this table due to incomplete data from the analysis output (i.e., comments disabled).

## Data Availability

The original contributions presented in this study are included in the article. Further inquiries can be directed to the corresponding author.

## Abbreviations

The following abbreviations are used in this manuscript:

AI	Artificial Intelligence
